# Comparison of Vital Sign Cutoffs to Identify Children With Major Trauma

**DOI:** 10.1001/jamanetworkopen.2023.56472

**Published:** 2024-02-16

**Authors:** Jillian K. Gorski, Pradip P. Chaudhari, Ryan G. Spurrier, Seth D. Goldstein, Suhail Zeineddin, Christian Martin-Gill, Robert J. Sepanski, Anne M. Stey, Sriram Ramgopal

**Affiliations:** 1 Division of Emergency Medicine, Ann & Robert H. Lurie Children’s Hospital of Chicago, Northwestern University Feinberg School of Medicine, Chicago, Illinois; 2 Division of Emergency and Transport Medicine, Department of Pediatrics, Children’s Hospital Los Angeles, Keck School of Medicine of the University of Southern California, Los Angeles; 3 Division of Pediatric Surgery, Department of Surgery, Children’s Hospital Los Angeles, Keck School of Medicine of the University of Southern California, Los Angeles; 4 Department of Surgery, Ann & Robert H. Lurie Children’s Hospital of Chicago, Northwestern University Feinberg School of Medicine, Chicago, Illinois; 5 Department of Emergency Medicine, University of Pittsburgh School of Medicine, Pittsburgh, Pennsylvania; 6 Department of Quality and Safety, Children’s Hospital of The King’s Daughters, Norfolk, Virginia; 7 Department of Pediatrics, Eastern Virginia Medical School, Norfolk; 8 Department of Surgery, Northwestern Memorial Hospital, Northwestern University Feinberg School of Medicine, Chicago, Illinois

## Abstract

**Importance:**

Vital signs are essential components in the triage of injured children. The Advanced Trauma Life Support (ATLS) and Pediatric Advanced Life Support (PALS) physiologic criteria are frequently used for trauma assessments.

**Objective:**

To evaluate the performance of ATLS and PALS criteria vs empirically derived criteria for identifying major trauma in children.

**Design, Setting, and Participants:**

This retrospective cohort study used 2021 American College of Surgeons Trauma Quality Improvement Program (TQIP) data contributed by US trauma centers. Included encounters involved pediatric patients (aged <18 years) with severe injury, excluding those who experienced out-of-hospital cardiac arrest, were receiving mechanical ventilation, or were transferred from another facility. Data were analyzed between April 9 and December 21, 2023.

**Exposure:**

Initial hospital vital signs, including heart rate, respiratory rate, and systolic blood pressure (SBP).

**Main Outcome and Measures:**

Major trauma, determined by the Standard Triage Assessment Tool, a composite measure of injury severity and interventions performed. Multivariable models developed from PALS and ATLS vital sign criteria were compared with models developed from the empirically derived criteria using the area under the receiver operating characteristic curve. Validation of the findings was performed using the 2019 TQIP dataset.

**Results:**

A total of 70 748 patients (median [IQR] age, 11 [5-15] years; 63.4% male) were included, of whom 3223 (4.6%) had major trauma. The PALS criteria classified 31.0% of heart rates, 25.7% of respiratory rates, and 57.4% of SBPs as abnormal. The ATLS criteria classified 25.3% of heart rates, 4.3% of respiratory rates, and 1.1% of SBPs as abnormal. Among children with all 3 vital signs documented (64 326 [90.9%]), PALS had a sensitivity of 88.4% (95% CI, 87.1%-89.3%) and specificity of 25.1% (95% CI, 24.7%-25.4%) for identifying major trauma, and ATLS had a sensitivity of 54.5% (95% CI, 52.7%-56.2%) and specificity of 72.9% (95% CI, 72.6%-73.3%). The empirically derived cutoff vital sign *z* scores had a sensitivity of 80.0% (95% CI, 78.5%-81.3%) and specificity of 48.7% (95% CI, 48.3%-49.1%) and area under the receiver operating characteristic curve of 70.9% (95% CI, 69.9%-71.8%), which was similar to PALS criteria (69.6%; 95% CI, 68.6%-70.6%) and greater than ATLS criteria (65.4%; 95% CI, 64.4%-66.3%). Validation using the 2019 TQIP database showed similar performance to the derivation sample.

**Conclusions and Relevance:**

These findings suggest that empirically derived vital sign criteria strike a balance between the sensitivity of PALS criteria and the specificity of ATLS criteria in identifying major trauma in children. These criteria may help to identify children at greatest risk of trauma-related morbidity and mortality.

## Key Points

**Question:**

What is the association between vital signs and major trauma in children?

**Findings:**

In this cohort study of 70 748 children, Advanced Trauma Life Support criteria had high specificity and Pediatric Advanced Life Support criteria had high sensitivity for classifying pediatric major trauma. Empirically derived vital sign criteria demonstrated an improved balance between sensitivity and specificity in the identification of pediatric major trauma.

**Meaning:**

These findings suggest that use of empirically derived vital sign cutoffs may improve the identification of major trauma in children, with the potential to assist with triage decisions and be used in clinical prediction models.

## Teaser

This cohort study compares the performance of Advanced Trauma Life Support and Pediatric Advanced Life Support criteria with empirically derived criteria for identifying pediatric major trauma.

## Introduction

Trauma is the leading cause of death for children in the US.^1^ An accurate assessment of injured children is essential for triage and disposition, as recognized by the American College of Surgeons (ACS).^2^ Nearly all decisions regarding the care of injured children are centered around physiologic criteria.^3,4,5,6,7^ Despite ACS guidelines on minimum criteria for the highest level of trauma activation, there is no consensus on which physiologic criteria should be used to define the need for trauma team activation.^8^ In a study evaluating criteria used by trauma centers for pediatric trauma activation, 92% used at least 1 physiologic criterion, with 72% including hypotension, 44% including heart rate, and 32% including respiratory rate.^9^

The ACS Advanced Trauma Life Support (ATLS) vital sign criteria are intended to assist frontline clinicians in identifying hypovolemic shock and respiratory failure.^2^ These criteria may have limited accuracy in identifying patients with shock.^10,11,12^ Similarly, the Pediatric Advanced Life Support (PALS) criteria provide another important means to classify vital signs for children with trauma in both the prehospital and in-hospital setting.^13,14^ Alternatively, vital sign classification using a robust, clinically sensible outcome measure may provide a more meaningful approach toward the diagnosis of severe injury. For patients experiencing trauma, this clinically important outcome is generally recognized to be major trauma, defined as a severe injury causing physiologic derangements or fatality.^15^ One newly derived measure of major trauma, the Standard Triage Assessment Tool (STAT), identifies acuity based on trauma interventions and injury severity.^16^ In this study, our aim was to identify the proportion of children (aged <18 years) with major trauma having abnormal vital signs based on PALS and ATLS criteria, empirically derive ranges for age-based vital signs for major trauma per STAT and validate these findings in a large sample of pediatric patients with trauma, and evaluate and compare the performance of these models using differing pediatric vital sign ranges for major trauma.

## Methods

### Data Source

We performed a retrospective cohort study using the 2021 ACS National Trauma Data Bank (NTDB) Trauma Quality Improvement Program (TQIP) Participant Use File (PUF). The TQIP PUF contains deidentified emergency and inpatient encounter data aggregated annually from hundreds of US hospitals that voluntarily contribute data to the NTDB. For validation of our findings, we used the 2019 TQIP PUF. Performance of this study was approved by the institutional review boards of the Ann & Robert H. Lurie Children’s Hospital of Chicago and ACS, with a waiver of the requirement of informed consent due to the deidentified nature of the study dataset. All methods were performed in accordance with the relevant guidelines and regulations of the ACS. This study adhered to the Transparent Reporting of a Multivariable Prediction Model for Individual Prognosis or Diagnosis (TRIPOD) guidelines. Detailed procedures are described in the eMethods in Supplement 1.

### Eligibility Criteria

We included all pediatric patient encounters within the NTDB. As the NTDB does not provide the exact age of infants (<12 months), we identified infants and estimated their age as a fraction of 1 year by using the inverse of the best-guess equation, an age-based weight estimation for infants.^17,18^ We excluded patients who experienced an out-of-hospital cardiac arrest, were receiving mechanical ventilation at the time of hospital presentation, were transferred from another facility, or lacked outcome data (ie, no calculable injury severity score). The first 2 exclusions were performed due to the limited value of vital signs in a patient experiencing cardiac arrest and lack of a natural respiration rate during mechanical ventilation. We excluded interfacility transfers as these patients may have been stabilized prior to their arrival.

### Data Acquisition, Exposures, and Outcomes

We acquired demographic, clinical, transport, trauma center, and outcome measure data. While TQIP identified patients by race and ethnicity, with heterogenous reporting definitions, we chose not to report or analyze race and ethnicity, given that race is a social construct without a physiologic basis and as there is limited utility of these social determinants within prediction models.

Our exposures of interest were initial heart rate, respiratory rate, and systolic blood pressure (SBP) after hospital arrival. Our outcome of interest was major trauma based on patient outcomes and interventions performed. We used STAT, a composite outcome measurement, to define major trauma as the presence of both high injury severity and need for intervention. This outcome requires meeting both Cribari Matrix (CM) and Need for Trauma Intervention (NFTI) criteria.^8,16^

### Statistical Analysis

We performed the data analysis between April 9 and December 21, 2023. We described the overall demographics of our sample, stratified by the presence of major trauma, and classified vital signs using the 2020 PALS^19^ and the 10th edition (2018) ATLS^2^ criteria. The ATLS guidelines contain only 1 limit to define abnormal vital signs (upper limits of heart rate and respiratory rate and lower limits of SBP), whereas PALS criteria provide upper and lower limits of each vital sign.

To generate optimal cutoffs of vital signs for age, we converted them to *z* scores using previously developed distributions for pediatric out-of-hospital emergencies^20,21^ and identified cutoffs for lower and higher vital signs by constructing receiver operating characteristic (ROC) curves for subsets of patients with vital signs below and above the median. We converted the *z* scores at these optimal cutoffs to vital sign thresholds within age groups of 0 to 3 months, 3 to 6 months, 6 to 9 months, 9 to 12 months, 1 to 3 years, 3 to 6 years, 6 to 9 years, 9 to 12 years, and more than 12 years. We selected these age groupings as they may be easier to deploy in practice, acknowledging that any system used to classify age ranges for vital signs is inherently arbitrary given the wide variability and individual differences in physiology within any selected grouping. We compared metrics of diagnostic accuracy (sensitivity, specificity, positive and negative predictive values, and positive and negative likelihood ratios) with 95% CIs for each vital sign criterion using the STAT outcome. Among children with all 3 vital signs documented, we evaluated the sensitivity and specificity of abnormal vital signs for each criterion, considering an encounter to have abnormal vital signs if at least 1 was abnormal. We constructed a multivariable model using the 3 vital sign criteria for the outcome of major trauma. We compared the area under the ROC curve (AUROC) of the models developed from PALS and ATLS criteria with the model developed from the empirically derived criteria.

To validate our findings, we applied the same inclusion criteria as for the primary sample using the 2019 TQIP dataset. We evaluated the performance of the 3 criteria. Among encounters with complete vital signs documentation, we evaluated the AUROCs with 95% CIs of the 3 multivariable models.

We also considered alternative outcome measures, including the CM, NFTI, and Need for Emergent Intervention Within 6 Hours (NEI-6).^22^ Analyses were performed using Stata, version 17.0 (StataCorp LLC) and the cutpointr, version 1.1.1^23^; gamlss, version 5.4-1^24^; and rms, version 6.2-0^25^ packages in R, version 4.3.2 (R Foundation for Statistical Computing). A 2-sided *P* < .05 was considered significant for differences between sets of criteria.

## Results

We included 70 748 pediatric encounters. The median (IQR) patient age was 11 (5-15) years, and 63.4% were male and 35.2% female. Detailed demographic information is provided in Table 1, and the patient inclusion flow diagram is provided in eFigure 1 in Supplement 1. Among the included patients, heart rate was documented in 99.7%, respiratory rate in 100%, and SBP in 91.1%. A total of 3223 patients (4.6%) met the STAT outcome measure for major trauma. The frequencies of outcomes and interventions used to derive NFTI, STAT, and NEI-6 are provided in eTable 1 in Supplement 1.

**Table 1.** Sample Demographics

Variable	No. (% of category)		
	All patients	Major trauma^a^	Not major trauma^a^
No.	70 748	3223	67 525
Age, median (IQR), y	11 (5-15)	14 (7-16)	10 (5-15)
Sex			
Male	44 830 (63.4)	2189 (67.9)	42 641 (63.2)
Female	24 890 (35.2)	989 (30.7)	23 901 (35.4)
Nonbinary	21 (<0.1)	1 (<0.1)	20 (<0.1)
Missing	1007 (1.4)	44 (1.4)	963 (1.4)
Payer type			
Government	32 685 (46.2)	1581 (49.1)	31 104 (46.1)
Private	31 214 (44.1)	1288 (40.0)	29 926 (44.3)
Self-pay	4106 (5.8)	206 (6.4)	3900 (5.8)
Not billed	77 (0.1)	7 (0.2)	70 (0.1)
Other	1363 (1.9)	64 (2.0)	1299 (1.9)
Missing	1303 (1.8)	77 (2.4)	1226 (1.8)
Mechanism			
Traffic related	18 377 (26.0)	1476 (45.8)	16 901 (25.0)
Other transportation^b^	7449 (10.5)	255 (7.9)	7194 (10.7)
Fall	24 712 (34.9)	328 (10.2)	24 384 (36.1)
Natural or environmental	2455 (3.5)	29 (0.9)	2426 (3.6)
Firearm	4094 (5.8)	670 (20.8)	3424 (5.1)
Cut or puncture	2243 (3.2)	50 (1.6)	2193 (3.3)
Struck by or against	6175 (8.7)	122 (3.8)	6053 (9.0)
Machinery	134 (0.2)	2 (0.1)	132 (0.2)
Overexertion	658 (0.9)	0	658 (1.0)
Drowning	11 (<0.1)	0	11 (<0.1)
Fire	7 (<0.1)	1 (<0.1)	6 (<0.1)
Other	1354 (1.9)	22 (0.7)	1332 (2.0)
Missing	3079 (4.4)	268 (8.3)	2811 (4.2)
Transport type			
Private vehicle	30 611 (43.3)	356 (11.1)	30 255 (44.8)
Ground	35 350 (50.0)	2310 (71.7)	33 040 (48.9)
Flight	4269 (6.0)	498 (15.5)	3771 (5.6)
Other^c^	371 (0.5)	53 (1.6)	318 (0.5)
Missing	147 (0.2)	6 (0.2)	141 (0.2)
Trauma center type			
Pediatric level I	19 112 (27.0)	839 (26.0)	18 273 (27.1)
Pediatric level II	9641 (13.6)	459 (14.2)	9182 (13.6)
Adult level I^d^	8862 (12.5)	637 (19.8)	8225 (12.2)
Adult level II or lower^d^	15 248 (21.6)	588 (18.2)	14 660 (21.7)
Not a trauma center	17 885 (25.3)	700 (21.7)	17 185 (25.5)
GCS category^e^			
≥9	66 085 (93.4)	2500 (77.6)	63 585 (94.2)
<9	1086 (1.5)	638 (19.8)	448 (0.7)
Missing	3577 (5.1)	85 (2.6)	3492 (5.1)
Emergency department disposition			
Admit	49 285 (69.7)	2942 (91.3)	46 343 (68.6)
Discharge	9398 (13.3)	10 (0.3)	9388 (13.9)
Transfer	11 476 (16.2)	224 (7.0)	11 252 (16.7)
Left against medical advice	68 (0.1)	0	68 (0.1)
Death	64 (0.1)	45 (1.4)	19 (<0.1)
Missing	457 (0.7)	2 (0.1)	455 (0.7)

Abbreviation: GCS, Glasgow Coma Score.

^a^ Per Standard Triage Assessment Tool trauma definition.

^b^ Other transportation includes pedal cyclist, pedestrian, and other nonspecified transportation modes.

^c^ Other transport included police transport and other nonspecified transport mode.

^d^ Adult trauma centers lacking pediatric verification.

^e^ Higher GCS scores indicate higher levels of consciousness.

### Comparison of Vital Signs Classification Using PALS and ATLS Criteria

**Figure 1.**
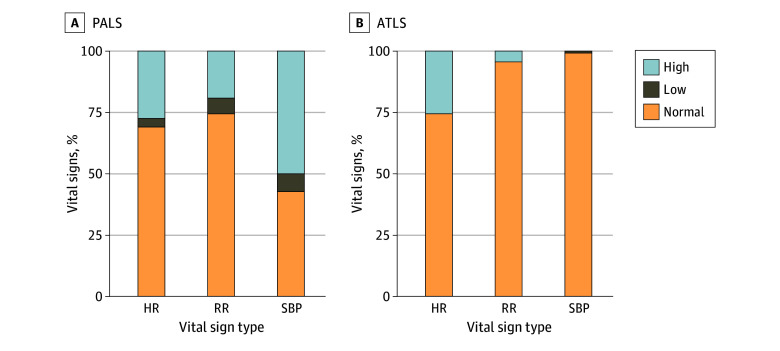
Classification of Vital Signs Using Differing Criteria Age-based vital signs were classified by Pediatric Advanced Life Support criteria as abnormal when they fell outside of the following ranges: heart rate (in beats per minute), 100 to 125 (age <28 days), 100 to 180 (28 days-1 year), 98 to 140 (1-3 years), 80 to 120 (4-6 years), 75 to 118 (6-12 years), and 60 to 100 (>12 years); respiratory rate (in breaths per minute), 30 to 53 (aged <1 year), 22 to 37 (1-2 years), 20 to 28 (3-5 years), 18 to 25 (6-11 years), and 12 to 20 (≥12 years); and systolic blood pressure (in mm Hg), 39 to 59 (aged 0-96 hours), 67 to 84 (3 days-1 month), 72 to 104 (1-12 months), 86 to 106 (1-2 years), 89 to 112 (3-5 years), 97 to 115 (6-9 years), 102 to 120 (10-12 years), and 110 to 131 (≥12 years).^8^ Age-based vital signs were classified by Advanced Trauma Life Support criteria as abnormal for heart rates (in beats per minute) greater than 160 (aged 0-12 months), 150 (1-2 years), 140 (3-5 years), 120 (6-12 years), and 100 (≥13 years); for respiratory rates (in breaths per minute) greater than 60 (aged 0-12 months), 40 (1-2 years), 35 (3-5 years), and 30 (≥6 years); and for systolic blood pressures (in mm Hg) lower than 60 (aged 0-12 months), 70 (1-2 years), 75 (3-5 years), 80 (6-12 years), and 90 (≥13 years). ATLS indicates Advanced Trauma Life Support; HR, heart rate; PALS, Pediatric Advance Life Support; RR, respiratory rate; SBP, systolic blood pressure.

The PALS criteria classified 31.0% of heart rates, 25.7% of respiratory rates, and 57.4% of SBPs as abnormal (Figure 1). The ATLS criteria classified 25.3% of heart rates, 4.3% of respiratory rates, and 1.1% of SBPs as abnormal. The presence of vital sign abnormalities by age with these 2 sets of criteria is presented in eFigure 2 in Supplement 1. Abnormally high vital signs occurred more frequently with PALS criteria compared with abnormal low vital signs measures.

### Association of Vital Signs With Major Trauma

**Figure 2.**
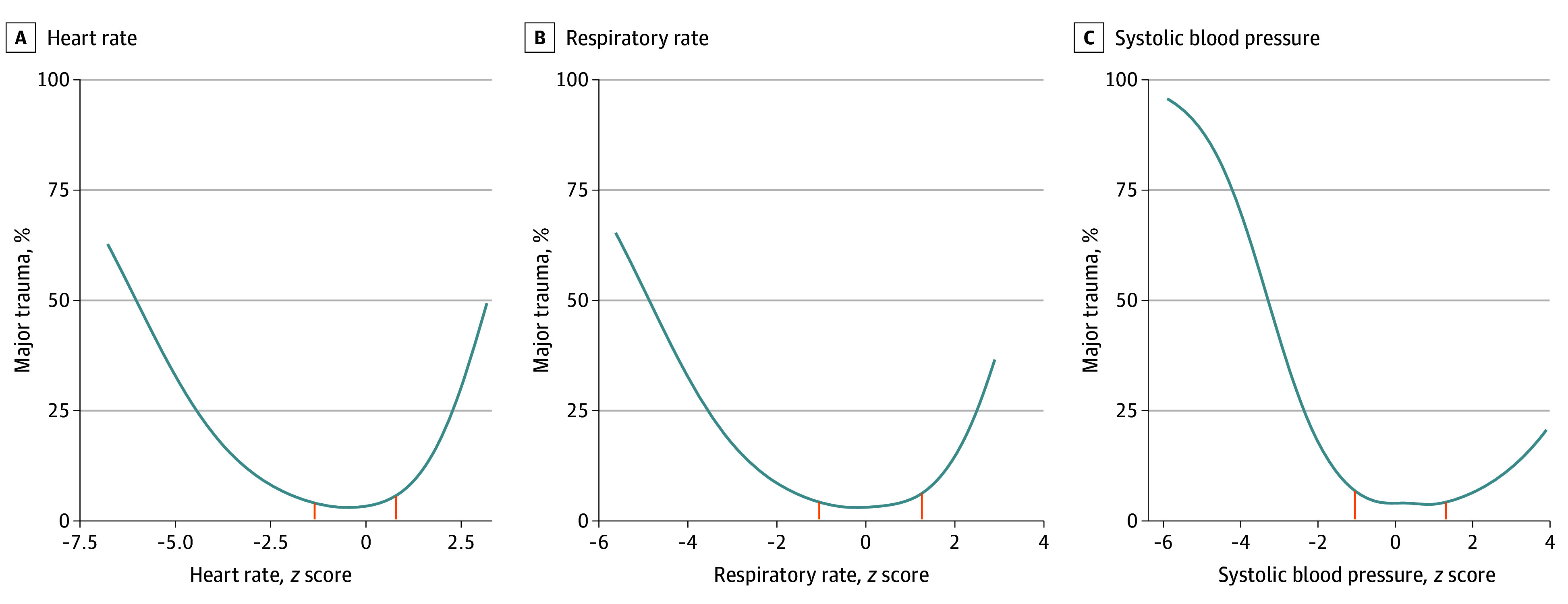
Association of Individual Vital Signs With Major Trauma, Using the Standard Triage Assessment Tool

All age-adjusted *z* scores of vital signs showed a U-shape association with major trauma (Figure 2). Evaluated as a continuous measure, calibration was generally satisfactory for all 3 vital signs at lower predicted probabilities of major trauma (eFigure 3 in Supplement 1). Cutoffs selected based on the ROC curve are provided in Table 2. The PALS vital signs criteria for heart rate and respiratory rate showed a blend between sensitivity and specificity for major trauma; in contrast, PALS SBP criteria favored sensitivity (65.1%; 95% CI, 63.4%-66.7%) at the expense of lower specificity (43.0%; 95% CI, 42.6%-43.4%) (Table 3). The ATLS criteria for heart rate showed lower sensitivity (47.6%; 95% CI, 45.9%-49.4%) but higher specificity (75.7%; 95% CI, 75.4%-76.0%) compared with PALS. The ATLS criteria for respiratory rate and SBP showed poor sensitivity (12.3% [95% CI, 11.1%-13.4%] and 10.0% [95% CI, 8.9%-11.1%], respectively) but high specificity (96.1% [95% CI, 96.0%-96.3%] and 99.3% [95% CI, 99.3%-99.4%], respectively). Based on empirically derived cutoffs, performance for heart rate, respiratory rate, and SBP showed gains in overall specificity (75.7% [95% CI, 75.3%-76.0%], 82.0% [95% CI, 81.7%-82.3%], and 81.4% [95% CI, 81.1%-81.7%], respectively) compared with PALS but at the cost of lower sensitivity (48.2% [95% CI, 46.5%-50.0%], 41.0% [95% CI, 39.3%-42.7%], and 38.5% [95% CI, 36.8%-40.2%], respectively). Compared with ATLS, these same cutoffs showed higher sensitivity but lower specificity. When using simplified cutoffs based on age group, performance was similar to using continuous age-based cutoffs.

**Table 2.** Optimal Cutoffs of Vital Signs by Age

Age group	Heart rate, beats/min	Respiratory rate, breaths/min	Systolic blood pressure, mm Hg
*z* Score for lower limit	−1.31	−1.01	−1.03
*z* Score for upper limit	0.84	1.28	1.32
0 to <3 mo	101-163	22-51	74-140
3 to <6 mo	109-160	23-46	81-127
6 to <9 mo	107-156	22-42	84-129
9 mo to <1 y	105-156	22-40	86-131
1 to <3 y	98-152	21-36	90-134
3 to <6 y	87-133	18-30	94-131
6 to <9 y	80-122	17-26	99-133
9 to <12 y	77-117	16-24	103-138
12 to <18 y	71-113	15-22	110-148

**Table 3.** Comparison of Empirically Derived Cutoffs of Vital Signs for an Outcome of Major Trauma With Pediatric Advanced Life Support (PALS) and Advanced Trauma Life Support (ATLS) Criteria

Vital sign criteria	Sensitivity, % (95% CI)	Specificity, % (95% CI)	PPV, % (95% CI)	NPV, % (95% CI)	PLR (95% CI)	NLR (95% CI)
**PALS**						
Heart rate	53.7 (51.9-55.4)	70.1 (69.7-70.4)	7.9 (7.5-8.3)	96.9 (96.8-97.1)	1.8 (1.7-1.9)	0.7 (0.6-0.7)
Respiratory rate	46.7 (45.0-48.4)	75.3 (75.0-75.6)	8.3 (7.9-8.7)	96.7 (96.6-96.9)	1.9 (1.8-2.0)	0.7 (0.7-0.7)
SBP	65.1 (63.4-66.7)	43.0 (42.6-43.4)	5.5 (5.3-5.8)	96.0 (95.8-96.2)	1.1 (1.1-1.2)	0.8 (0.8-0.9)
**ATLS**						
Heart rate	47.6 (45.9-49.4)	75.7 (75.4-76.0)	8.6 (8.2-9.0)	96.8 (96.6-97.0)	2.0 (1.9-2.0)	0.7 (0.7-0.7)
Respiratory rate	12.3 (11.1-13.4)	96.1 (96.0-96.3)	13.1 (11.9-14.3)	95.8 (95.7-96.0)	3.2 (2.9-3.5)	0.9 (0.9-0.9)
SBP	10.0 (8.9-11.1)	99.3 (99.3-99.4)	43.4 (39.8-47.1)	95.6 (95.4-95.7)	14.9 (13.0-17.2)	0.9 (0.9-0.9)
**Empirically derived using *z* scores**						
Heart rate	48.8 (47.0-50.5)	75.1 (74.7-75.4)	8.5 (8.1-9.0)	96.8 (96.7-97.0)	2.0 (1.9-2.0)	0.7 (0.7-0.7)
Respiratory rate	45.8 (44.1-47.5)	79.0 (78.6-79.3)	9.4 (9.0-9.9)	96.8 (96.7-97.0)	2.2 (2.1-2.3)	0.7 (0.7-0.7)
SBP	41.0 (39.2-42.7)	79.3 (79.0-79.6)	9.2 (8.8-9.7)	96.3 (96.1-96.5)	2.0 (1.9-2.1)	0.7 (0.7-0.8)
**Empirically derived using simplified age-based cutoffs**						
Heart rate	48.2 (46.5-50.0)	75.7 (75.3-76.0)	8.6 (8.2-9.1)	96.8 (96.7-97.0)	2.0 (1.9-2.1)	0.7 (0.7-0.7)
Respiratory rate	41.0 (39.3-42.7)	82.0 (81.7-82.3)	9.8 (9.3-10.3)	96.7 (96.5-96.8)	2.3 (2.2-2.4)	0.7 (0.7-0.7)
SBP	38.5 (36.8-40.2)	81.4 (81.1-81.7)	9.6 (9.1-10.2)	96.3 (96.1-96.4)	2.1 (2.0-2.2)	0.8 (0.7-0.8)

Abbreviations: NLR, negative likelihood ratio; NPV, negative predictive value; PLR, positive likelihood ratio; PPV, positive predictive value; SBP, systolic blood pressure.

Among encounters with all 3 vital signs documented (64 326 [90.9%]), 3144 (4.9%) met the STAT outcome for major trauma. Within this sample, PALS, ATLS, and the empirically derived criteria identified 48 609 (75.6%), 18 275 (28.4%), and 33 910 (52.7%) children, respectively, with at least 1 abnormal vital sign. The PALS criteria had a sensitivity of 88.4% (95% CI, 87.1%-89.3%) and a specificity of 25.1% (95% CI, 24.7%-25.4%). The ATLS criteria had a sensitivity of 54.5% (95% CI, 52.7%-56.2%) and a specificity of 72.9% (95% CI, 72.6%-73.3%). The empirically derived cutoffs had a sensitivity of 80.0% (95% CI, 78.5%-81.3%) and a specificity of 48.7% (95% CI, 48.3%-49.1%). All vital sign criteria showed a high negative predictive value (PALS, 97.6% [95% CI, 97.4%-97.9%]; ATLS, 96.9% [95% CI, 96.7%-97.0%]; empirically derived cutoffs, 97.9% [95% CI, 97.8%-98.1%]). Univariable and multivariable odds ratios of each set of criteria are provided in eTable 2 in Supplement 1. The AUROC for a model using the empirically derived criteria (70.9%; 95% CI, 69.9%-71.8%) was similar to the AUROC for a model using PALS criteria (69.6%; 95% CI, 68.6%-70.6%; *P* = .001 for the difference) and was greater than the model derived from ATLS criteria (65.4%; 95% CI, 64.4%-66.3%; *P* < .001 for the difference) (eTable 4 in Supplement 1).

### Validation

A total of 1 097 190 encounters were present in the 2019 TQIP PUF file, of which 72 858 (6.6%) were included for validation. Major trauma defined by STAT was documented in 2814 encounters (3.9%). The application of the PALS, ATLS, and empirically derived criteria to this sample for an outcome of major trauma produced similar findings to those described in the derivation sample. Differences in accuracy were similar when using the empirically derived vital sign cutoffs (eTable 3 in Supplement 1). The AUROCs within the validation sample were similar to those reported among the derivation sample (eTable 4 in Supplement 1).

### Additional Analyses

The CM outcome occurred in 6773 encounters (9.5%), the NFTI outcome occurred in 10 290 encounters (14.5%), and the NEI-6 outcome occurred in 12 428 encounters (17.6%). Splined univariable analyses of empirically derived vital signs with these outcomes are provided in eFigure 4 in Supplement 1. All displayed a similar appearance to the primary analysis performed using the outcome of STAT, with differing baseline probabilities reflecting the baseline prevalence of each outcome. Vital sign ranges using these alternative outcomes were more extreme for heart rate (eTable 5 in Supplement 1). The lower limits of respiratory rate were similar with each cutoff. The NFTI and NEI-6 had similar upper ranges of respiratory rate compared with the primary analysis, whereas the upper limit of respiratory rate was lower for CM. The lower limit of SBP was similar for all 4 outcomes, with differences observed in the upper limit of SBP. Performance metrics for each outcome when using PALS, ATLS, and empirically derived vital signs criteria were similar to findings reported in the primary analysis (eTable 6 in Supplement 1).

## Discussion

We used a nationally representative trauma registry to generate and validate ranges of vital signs to optimally correlate with the presence of major trauma in children. These empirically derived vital sign cutoffs had greater sensitivity for identifying major trauma than ATLS criteria, particularly for respiratory rate and SBP, and were more specific for identifying major trauma than PALS criteria, particularly for SBP. Performance was comparable between the derivation and validation samples. Prospectively validated, these empirically derived vital sign criteria for injured children may provide an opportunity for more accurate prehospital and early hospital trauma triage, potentially optimizing hospital resource utilization.

We provide a data-driven approach by using vital signs to identify children with major trauma. Our study supports the use of physiologic criteria in trauma risk assessment and is of great relevance given the lack of consensus for vital sign norms among injured children.^9^ The use of outcomes as a basis for determining vital sign criteria may help to better identify children needing intervention in prehospital and early hospital settings, where alterations in vital signs can be secondary to pain and anxiety. Notably, all vital sign criteria showed a high negative predictive value for major trauma, suggesting that vital signs may be useful in identifying children who do not have this outcome. When considering all vital signs in aggregate, empirically derived vital signs criteria showed superior performance to PALS, which had low specificity, and to ATLS criteria, which had low sensitivity. Used as a continuous predictor, these findings could be incorporated into prediction modeling applications when combined with additional prehospital and hospital-based variables to ensure the appropriateness of triage in pediatric trauma. The AUROC of a model built using the empirically derived vital signs criteria showed statistically significant improvement over the PALS and ATLS criteria for identifying major trauma in children.

Our findings contribute to the existing literature evaluating age-based associations with vital signs and trauma. A previous study of the NTDB evaluated associations between heart rate and mortality among children, seeking to identify ranges with the lowest odds of mortality.^26^ While the heart rate ranges for reduced mortality risk in that study are similar to those reported in the present analysis, we identified cutoffs that may improve diagnostic accuracy. Several studies have reported significant findings of systolic hypotension using differing age-based criteria for the prediction of mortality and other clinically important outcomes, including traumatic brain injury and shock, among pediatric patients with trauma.^27,28,29,30^ Our approach corroborates other data-driven approaches to physiologic indicators of morbidity in pediatric trauma, such as the pediatric shock index.^31^

The ATLS vital signs criteria showed high specificity but poor sensitivity for major trauma, particularly for respiratory rate and SBP. Triage algorithms have largely focused on sensitivity, with the goal of identifying all potential patients with major trauma who may benefit from transfer to a higher level of care or who may require urgent interventions. Nevertheless, the benefits of higher sensitivity may be adversely affected by criteria that overclassify a large proportion of children as having major trauma. Our study joins a body of work among injured adults that suggests that ATLS criteria may be suboptimal in identifying patients with a higher risk of morbidity,^10,11,32^ which may be partly due to the 1-sided nature of the ATLS criteria and may miss opportunities to classify patients at risk of major trauma based on hypertension, bradypnea, or bradycardia.^2^ Characterizing patients with vital sign extremes is particularly important in children with neurotrauma, who have a distinct pathophysiology of vital sign derangements compared with those with hemorrhagic shock. Ultimately, the low sensitivity of ATLS criteria in this study suggests a need to consider 2-sided vital sign ranges in activation criteria for injured children to avoid misclassifying children with the highest interventional need.

Our empirically derived vital signs were most comparable with PALS-based criteria but with a lower proportion of patients classified as having abnormal vital signs (75.6% with PALS vs 52.7% with empirically derived cutoffs). Previously published work evaluating empirically derived vital sign criteria for children experiencing out-of-hospital pediatric emergencies indicated improved sensitivity over PALS for identifying children requiring prehospital interventions.^20,21^ Conversely, our findings show that these cutoffs have improved specificity but poorer sensitivity compared with PALS for identifying children with major trauma. While sensitivity remains arguably the more important factor for screening children with major trauma, the AUROC of the empirically derived vital sign cutoffs showed a statistically significant improvement over PALS, which may be associated with the more representative underlying population used to create these cutoffs (ie, out-of-hospital emergencies rather than healthy children). These findings warrant follow-up through prospective validation. Future work may seek to identify the accuracy of these vital sign criteria, their dynamic changes over time, and their association with other physiologic measures. Additionally, research is needed to determine which other contextual characteristics, such as mechanism of injury, in conjunction with physiologic criteria may help to identify which children are at risk of major trauma and, by association, trauma-related mortality and morbidity. Future work should also include engagement with partners to identify how these criteria may be used to optimize care. One possible application includes the implementation of models to risk stratify pediatric patients with trauma in the prehospital setting, identifying those with the greatest need for acute interventions and assisting with triage to the optimal site of care.

### Limitations

Our findings are subject to limitations. This analysis was performed using an existing dataset that may have errors in coding and data abstraction. We were required to provide estimates for age in infants. Not all children had all vital signs reported, and a small proportion lacked outcome data. We were unable to identify all relevant outcomes for children transferred to a higher level of care. Furthermore, procedural interventions, intrinsic to the STAT and NFTI outcomes of major trauma, can be variable across institutions, limiting external validity. We excluded out-of-hospital transfers, which represent an important population of children evaluated at tertiary care trauma centers. Future work may benefit from evaluating the association of vital signs among patients who may have been partially stabilized at a receiving institution. Many of these children would likely have been initially evaluated at nonparticipating NTDB hospitals, including hospitals with less pediatric trauma exposure or rural hospitals, leading to potentially skewed exclusion.

## Conclusions

In this cohort study, we identified pediatric trauma victims with abnormal vital signs using empirically-derived vital sign cutoffs and found that these abnormal vital signs were associated with major trauma in these patients. These vital sign cutoffs, previously studied in the prehospital setting, may improve the care of injured children by providing a set of standards that appropriately balances sensitivity and specificity in identifying children at risk of trauma-related morbidity. These criteria, when combined with other contextual characteristics, could be used in field trauma triage as well as early trauma team activation for ensuring appropriate resource mobilization in the care of injured children.

## Notes

Supplement 1.
eMethods.

eReferences
**eFigure 1.** Patient Inclusion **eFigure 2.** Presence of Abnormal Vital Signs by Age When Using the Pediatric Advanced Life Support and Advanced Trauma Life Support Guidelines **eFigure 3.** Calibration Plots of Univariable Predictors of Major Trauma When Using Age-Based *z* Scores for Heart Rate, Respiratory Rate, and Systolic Blood Pressure **eFigure 4.** Association of Individual Vital Signs With Critical Illness With Different Outcomes **eTable 1.** Frequency of Outcomes and Interventions Performed Used to Derive the Study Outcomes **eTable 2.** Univariable and Multivariable Models of Vital Sign Criteria **eTable 3.** External Validation of Vital Sign Criteria (Including the Empirically Derived Vital Sign Criteria, Derived From the 2021 Trauma Quality Improvement Program [TQIP] Dataset) to the 2019 TQIP Dataset for an Outcome of Major Trauma **eTable 4.** Areas Under the Receiver Operating Characteristic Curve (AUROCs) With 95% CIs for Multivariable Models in the Derivation (2021 TQIP) and Validation (2019 TQIP) Samples **eTable 5.** Alternative Cutoffs for Vital Signs Obtained When Using Different Outcome Measures **eTable 6.** Diagnostic Accuracy of Cutoffs (Derived From Different Outcomes) in Addition to PALS and ATLS Criteria

Supplement 2.
Data Sharing Statement
