# Error in Results and eTables 3 and 4

**DOI:** 10.1001/jamanetworkopen.2024.32946

**Published:** 2024-08-13

In the Original Investigation titled “Comparison of Vital Sign Cutoffs to Identify Children With Major Trauma,”^1^ published online February 16, 2024, an error in applying exlusion criteria for the validation sample resulted in incorrect measures of accuracy for the sample in the Results and in eTables 3 and 4 in Supplement 1. This article has been corrected.^1^
