# Supplementary material for: Comparison of Vital Sign Cutoffs to Identify Children With Major Trauma
Source: JAMA Netw Open. 2024 Feb 16;7(2):e2356472. doi: 10.1001/jamanetworkopen.2023.56472 (PMC10873773; doi:10.1001/jamanetworkopen.2023.56472)
Supplement: Supplement 1. — eMethods. eReferences eFigure 1. Patient Inclusion eFigure 2. Presence of Abnormal Vital Signs by Age When Using the Pediatric Advanced Life Support and Advanced Trauma Life Support Guidelines eFigure 3. Calibration Plots of Univariable Predictors of Major Trauma When Using Age-Based z Scores for Heart Rate, Respiratory Rate, and Systolic Blood Pressure eFigure 4. Association of Individual Vital Signs With Critical Illness With Different Outcomes eTable 1. Frequency of Outcomes and Interventions Performed Used to Derive the Study Outcomes eTable 2. Univariable and Multivariable Models of Vital Sign Criteria eTable 3. External Validation of Vital Sign Criteria (Including the Empirically Derived Vital Sign Criteria, Derived From the 2021 Trauma Quality Improvement Program [TQIP] Dataset) to the 2019 TQIP Dataset for an Outcome of Major Trauma eTable 4. Areas Under the Receiver Operating Characteristic Curve (AUROCs) With 95% CIs for Multivariable Models in the Derivation (2021 TQIP) and Validation (2019 TQIP) Samples eTable 5. Alternative Cutoffs for Vital Signs Obtained When Using Different Outcome Measures eTable 6. Diagnostic Accuracy of Cutoffs (Derived From Different Outcomes) in Addition to PALS and ATLS Criteria [file jamanetwopen-e2356472-s001.pdf]

Supplemental Online Content

Gorski JK, Chaudhari PP, Spurrier RG, et al. Comparison of vital sign cutoffs to identify children with major trauma. *JAMA Netw Open*. 2021;7(2):e2356472. doi:10.1001/jamanetworkopen.2023.56472

eMethods

eReferences

eFigure 1. Patient Inclusion

eFigure 2. Presence of Abnormal Vital Signs by Age When Using the Pediatric Advanced Life Support and Advanced Trauma Life Support Guidelines

eFigure 3. Calibration Plots of Univariable Predictors of Major Trauma When Using Age-Based *z* Scores for Heart Rate, Respiratory Rate, and Systolic Blood Pressure

eFigure 4. Association of Individual Vital Signs With Critical Illness With Different Outcomes

eTable 1. Frequency of Outcomes and Interventions Performed Used to Derive the Study Outcomes

eTable 2. Univariable and Multivariable Models of Vital Sign Criteria

eTable 3. External Validation of Vital Sign Criteria (Including the Empirically Derived Vital Sign Criteria, Derived From the 2021 Trauma Quality Improvement Program [TQIP] Dataset) to the 2019 TQIP Dataset for an Outcome of Major Trauma

eTable 4. Areas Under the Receiver Operating Characteristic Curve (AUROCs) With 95% CIs for Multivariable Models in the Derivation (2021 TQIP) and Validation (2019 TQIP) Samples

eTable 5. Alternative Cutoffs for Vital Signs Obtained When Using Different Outcome Measures

eTable 6. Diagnostic Accuracy of Cutoffs (Derived From Different Outcomes) in Addition to PALS and ATLS Criteria

This supplemental material has been provided by the authors to give readers additional information about their work.

© 2024 Gorski JK et al. *JAMA Network Open.*

eMethods

Data acquisition:

We acquired the following from each encounter: demographics including age (in years), weight (in kg), sex (male, female, non-binary, or missing) and payer (government, private, self-pay, not billed, other, missing); clinical characteristics including emergency department (ED) vital signs (heart rate, respiratory rate, and systolic blood pressure), mechanism, type of transport (private vehicle, ground transport, flight, other, and missing), type of trauma center (pediatric level 1, pediatric level 2, adult level 1, adult level 2 or lower, and not a trauma center), and Glasgow Coma Score (<9, ≥9, and missing); and outcome measures, including ED disposition and data required to derive major trauma scores. Missing categorical data were defined in their own category, without imputation. Data required to derive major trauma scores included abbreviated injury scale (AIS) data, from which Injury Severity Score was derived; provision of packed red blood cells; ED and hospital length of stay; use of the operating room, interventional radiology, or the intensive care unit; performance of mechanical ventilation, chest tube placement, or central line placement; and in-hospital mortality.

Operationalization of the Standard Triage Assessment Tool (STAT) outcome:

The STAT major trauma outcome is met when both of the following individual criteria are met: the Cribari Matrix (CM) and Need for Trauma Intervention (NFTI).[1] CM is defined as an ISS of >15 (maximum 75), calculated from individual AIS data.[2] NFTI is defined as the presence of any of the following interventions: administration of packed red blood cells within 4 hours of hospital arrival, transfer from the ED to the operating room within 90 minutes of hospital arrival, discharge to interventional radiology, discharge to the intensive care unit with an intensive care unit length of stay of ≥72 hours, performance of nonprocedural mechanical ventilation within 72 hours of hospital arrival, or mortality within 60 hours.[3,4] Adapting these criteria for the National Trauma Data Bank, we defined red blood cell administration as the provision of at least 300 milliliters of packed red blood cells, at least 500 milliliters of whole blood, or at least 10 milliliters per kilogram of packed red blood cells and/or whole blood. The interventional radiology criteria included angiography and was modified to identify all patients who underwent this procedure within 24 hours of ED arrival. The intensive care unit and mortality criteria were modified to use a cutoff of 3 days if length of stay in hours was unavailable.

Evaluation of the association of vital signs with major trauma:

To visualize the overall association of continuously measured age-adjusted vital signs with major trauma, we performed univariable binary logistic regression for each vital sign using a linear tail-restricted cubic spline function with five knots selected using maximum likelihood estimation, with the first and last knot set at the 5th and 95th percentiles. We constructed calibration curves of these univariable models from Z-scores to visually inspect the model across the range of predicted probabilities, with smoothing performed using a Locally Estimated Scatterplot Smoothing function.

Development of cutpoints:

We sought to develop dichotomous cutoffs in this evaluation given the complexity of interpreting tiered age-based cutoffs (such as those used within early warning system scores) and because the establishment of vital sign ranges is most reflective of how these criteria are used in practice. We separately identified cutpoints for each vital sign for their high and low values by analyzing patients with a high vital sign value (e.g., Z-scored vital sign >0) and a low vital sign (Z-scored vital sign <0) separately. This approach allowed us to identify separate criteria for these ranges rather than cutpoints equidistant from the Z score of 0. To identify the predictive value of cutoffs using empirically-derived Z-scores, we identified the optimal cutoffs for a high Z-score for major trauma by constructing a receiver operator characteristic curve for the subset of encounters having a Z-score for these respective values >0 and calculating the Youden Index. We then identified the optimal lower cutoff by using a similar procedure for Z-scores <0.

Multivariable model development:

To compare the overall performance of the differing age-based vital signs criteria, we constructed multivariable binary logistic regression models using vital signs for an outcome of major trauma. When using the Pediatric Advanced Life Support (PALS) and empirically-derived vital signs criteria, a vital sign was classified as being “High,” “Low,” or “Normal”; when using Advanced Trauma Life Support (ATLS) criteria, values were dichotomized. We described the performance of each model using odds ratios with 95% confidence intervals (CI). We calculated the area under the receiver operator characteristic curve (AUROC) with 95% CI. We compared the AUROC of the PALS and ATLS criteria to the empirically-derived criteria use the DeLong method, bootstrapped over 1,000 iterations.

© 2024 Gorski JK et al. *JAMA Network Open.*

Additional analyses:

We derived potential vital signs cutoffs using alternative outcome measures, including the CM, NFTI, and Need for Need for Emergent Intervention within 6 hours (NEI-6). The CM and NFTI definitions are provided above. The NEI-6 is comprised of the following: provision of 5 units of packed red blood cells within 4 hours, emergent trauma bay intubation or angiography; performance of emergent brain intervention, central line placement, or chest tube placement; or use of the operating room within 6 hours of hospital arrival. Using each outcome, we performed univariable analyses for each vital sign, evaluated optimal cutoffs, and compared these to PALS and ATLS vital sign criteria.

eReferences

1. Shahi N, Phillips R, Rodenburg C, et al. Combining Cribari matrix and Need For Trauma Intervention (NFTI) to accurately assess undertriage in pediatric trauma. *Journal of Pediatric Surgery*. 2021;56(8):1401-1404.
2. American College of S. Resources for Optimal Care of the Injured Patient (2022 Standards). 2022.
3. Roden-Foreman JW, Rapier NR, Foreman ML, et al. Rethinking the definition of major trauma: The need for trauma intervention outperforms Injury Severity Score and Revised Trauma Score in 38 adult and pediatric trauma centers. *Journal of trauma and acute care surgery*. 2019;87(3):658-665.
4. Roden-Foreman JW, Rapier NR, Yelverton L, Foreman ML. Asking a better question: development and evaluation of the need for trauma intervention (NFTI) metric as a novel indicator of major trauma. *Journal of trauma nursing*. 2017;24(3):150-157.

© 2024 Gorski JK et al. *JAMA Network Open.*

eFigure 1. Patient Inclusion

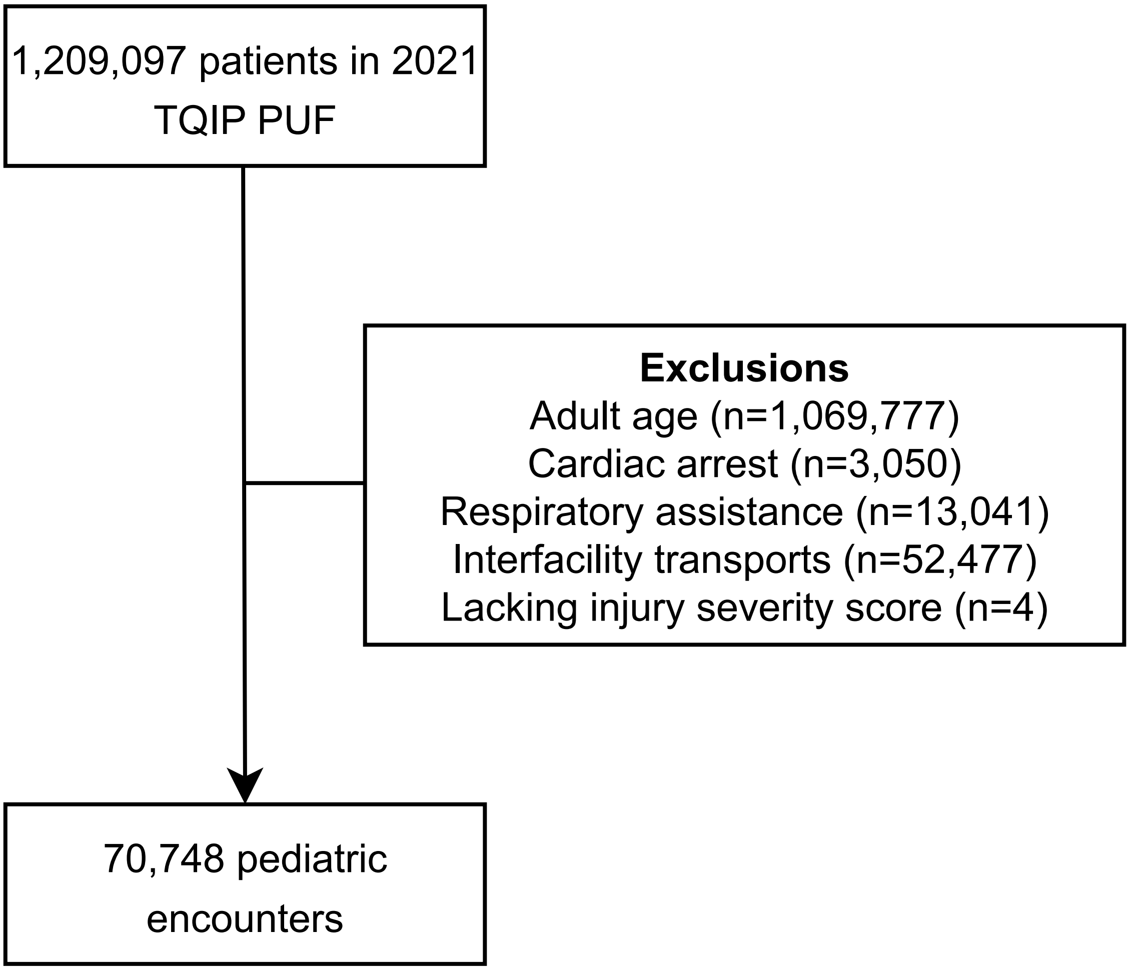

TQIP, Trauma Quality Improvement Program; PUF, Participant Use File

© 2024 Gorski JK et al. *JAMA Network Open.*

eFigure 2. Presence of Abnormal Vital Signs by Age When Using the Pediatric Advanced Life Support (PALS; A-C) and Advanced Trauma Life Support (ATLS; D-F) Guidelines. Note that Y-axis ranges for PALS vital signs and ATLS vital signs differ due to the lower proportion of patients with ATLS vital signs with abnormal vital signs.

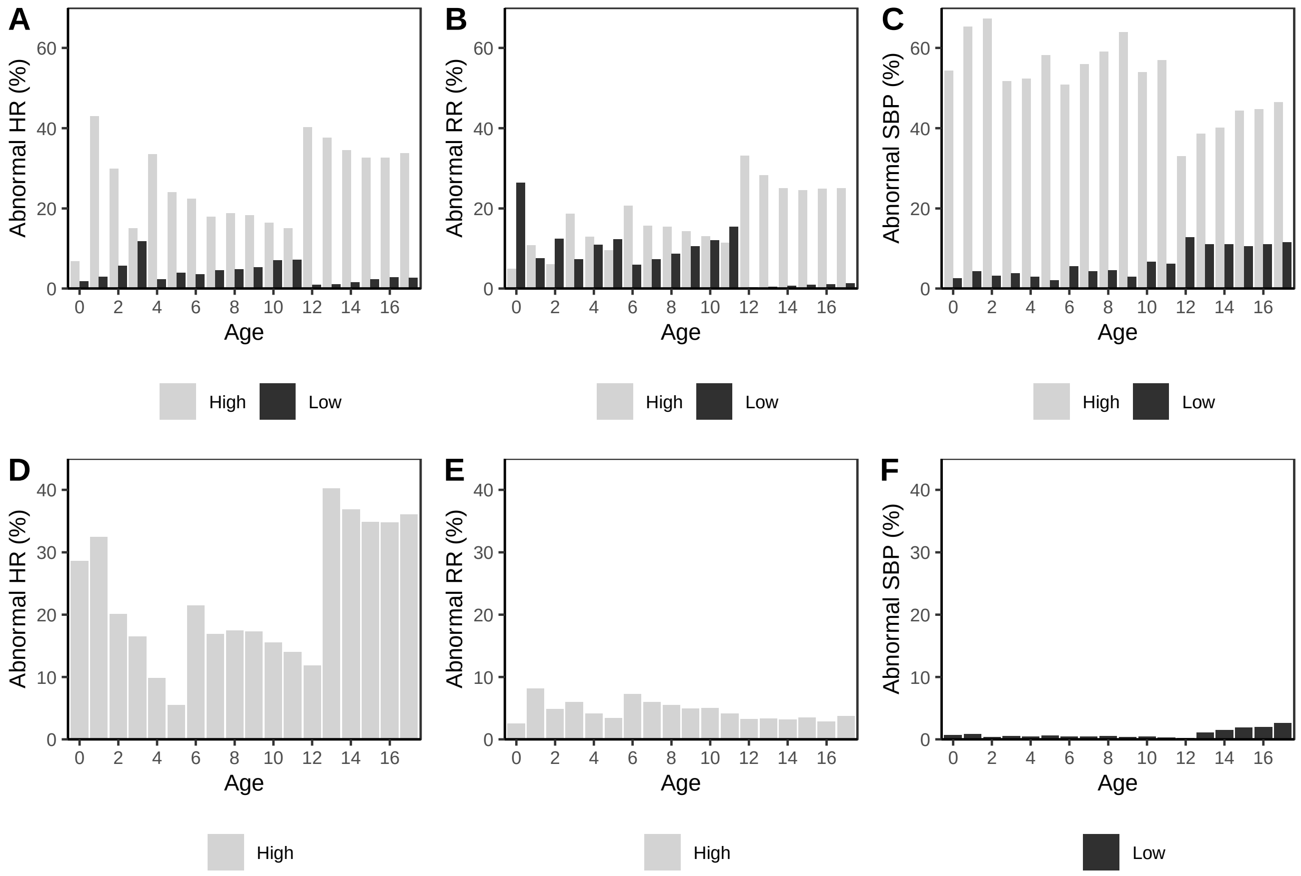

HR, heart rate; RR, respiratory rate; SBP, systolic blood pressure

© 2024 Gorski JK et al. *JAMA Network Open.*

eFigure 3. Calibration Plots of Univariable Predictors of Major Trauma When Using Age-Based *z* Scores for (A) heart rate, (B) respiratory rate, and (C) systolic blood pressure. These plots assess the association between the predicted probability (X-axis) to the actual probability (Y axis) of outcome, with ideal calibration representing a line with slope 1 and X intercept at the 0. An ideal test demonstrates perfect calibration between an actual and predicted probability of mortality (intercept of 0, and slope of 1).

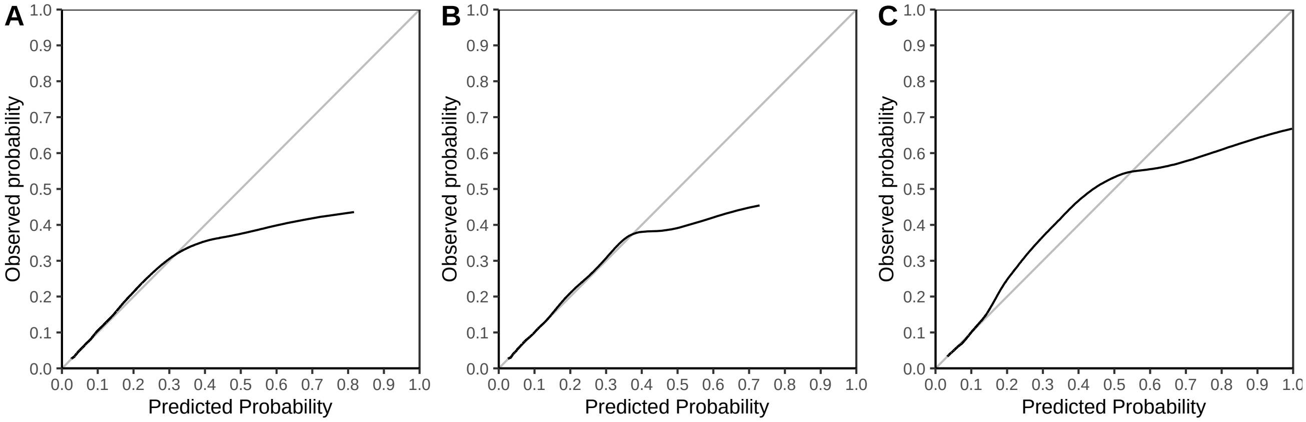

© 2024 Gorski JK et al. *JAMA Network Open.*

eFigure 4. Association of Individual Vital Signs With Critical Illness With Different Outcomes. The primary outcome used was the Standard Triangle Assessment Tool (STAT). Alternative outcome measures include the Cribari Matrix (CM), Need for Emergent Intervention within 6 hours (NEI6), and Need for Trauma Intervention (NFTI).

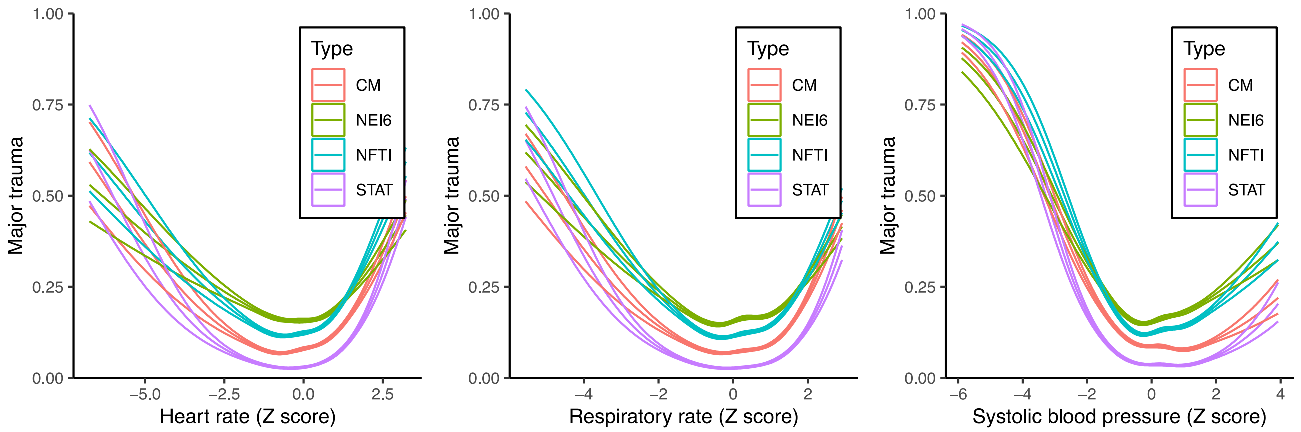

© 2024 Gorski JK et al. *JAMA Network Open.*

eTable 1. Frequency of Outcomes and Interventions Performed Used to Derive the Study Outcomes

Intervention	Count (% of cohort)
Transfusion within 4 hours	3,000 (4.2)
ED to OR within 90 minutes	3,374 (4.8)
ED to interventional radiology	206 (0.3)
ED to ICU with > 72 hour length of stay	3,989 (5.6)
Endotracheal intubation within 72 hours	2,264 (3.2)
Death within 60 hours of arrival	170 (0.2)
Central line placement within 6 hours	319 (0.5)
Chest tube placement within 6 hours	992 (1.4)
Brain/ICP intervention within 6 hours	76 (0.1)

ED, Emergency department; OR, operating room; ICU, intensive care unit; ICP, intracranial pressure

© 2024 Gorski JK et al. *JAMA Network Open.*

eTable 2. Univariable and Multivariable Models of Vital Sign Criteria. Coefficients and intercept terms apply to the multivariable model.

	Univariable OR (95% CI)	Multivariable OR (95% CI)	Coeff
**PALS (intercept= -3.75)**			
**Heart rate**			
Normal range	Ref	Ref	
High	2.67 (2.48-2.87)	2.26 (2.09-2.44)	0.82
Low	2.61 (2.23-3.05)	2.40 (2.04-2.82)	0.88
**Respiratory rate**			
Normal range	Ref	Ref	
High	2.90 (2.68-3.13)	2.34 (2.16-2.54)	0.85
Low	1.80 (1.57-2.07)	1.93 (1.67-2.22)	0.65
**Systolic blood pressure**			
Normal range	Ref	Ref	
High	0.99 (0.92-1.08)	0.92 (0.85-1.00)	-0.08
Low	4.39 (3.98-4.84)	3.94 (3.56-4.35)	1.37
**ATLS (intercept=-3.43)**			
**Heart rate**			
Normal range	Ref	Ref	
High	2.79 (2.59-3.00)	2.41 (2.24-2.60)	0.88
**Respiratory rate**			
Normal range	Ref	Ref	
High	3.38 (3.01-3.79)	2.46 (2.18-2.78)	0.90
**Systolic blood pressure**			
Normal range	Ref	Ref	
Low	16.41 (14.09-19.11)	12.94 (11.04-15.17)	2.46
**Empirically-derived criteria (intercept=-3.80)**			
**Heart rate**			
Normal range	Ref	Ref	
High	3.33 (3.08-3.61)	2.75 (2.54-2.99)	1.01
Low	2.00 (1.78-2.24)	1.88 (1.67-2.12)	0.63
**Respiratory rate**			
Normal range	Ref	Ref	
High	3.33 (3.08-3.61)	2.68 (2.47-2.92)	0.99
Low	2.34 (2.05-2.66)	2.26 (1.98-2.58)	0.82
**Systolic blood pressure**			
Normal range	Ref	Ref	
High	1.67 (1.50-1.86)	1.41 (1.26-1.58)	0.35
Low	4.14 (3.79-4.52)	3.84 (3.51-4.20)	1.34

PALS, Pediatric Advanced Life Support; ATLS, Advanced Trauma Life Support; CI, confidence interval; Coef, coefficient

© 2024 Gorski JK et al. *JAMA Network Open.*

eTable 3. External Validation of Vital Sign Criteria (Including the Empirically Derived Vital Sign Criteria, Derived From the 2021 Trauma Quality Improvement Program [TQIP] Dataset) to the 2019 TQIP Dataset for an Outcome of Major Trauma. Numbers in parenthesis represent 95% confidence intervals.

Vital sign	Sens	Spec	PPV	NPV	PLR	NLR
**PALS vital signs criteria**						
HR	53.2 (51.3-55.0)	71.3 (71.0-71.7)	6.9 (6.6-7.3)	97.4 (97.3-87.6)	1.9 (1.8-1.9)	0.7 (0.6-0.7)
RR	45.0 (43.1-48.8)	77.0 (76.7-77.3)	7.3 (6.9-7.7)	97.2 (97.1-97.3)	2.0 (1.9-2.0)	0.7 (0.7-0.7)
SBP	64.3 (62.5-66.1)	43.6 (43.2-44.0)	4.7 (4.5-74.9)	96.6 (96.3-96.8)	1.1 (1.1-1.2)	0.8 (0.8-0.9)
**ATLS vital signs criteria**						
HR	45.1 (43.3-47.0)	77.8 (77.5-78.1)	7.6 (7.2-8.0)	97.2 (97.1-97.4)	2.0 (2.0-2.1)	0.7 (0.7-0.7)
RR	11.9 (10.7-13.1)	96.2 (96.1-96.4)	11.3 (10.2-12.5)	96.5 (96.3-96.6)	3.2 (2.8-3.5)	0.9 (0.9-0.9)
SBP	9.9 (8.8-11.1)	99.4 (99.4-99.5)	42.7 (38.8-46.6)	96.2 (96.1-96.4)	17.1 (14.7-19.9)	0.9 (0.9-0.9)
**Empirically-derived vital signs using Z score**						
HR	47.9 (46-49.7)	74.9 (74.6-75.2)	7.1 (6.8-7.5)	97.3 (97.1-97.4)	1.9 (1.8-2.0)	0.7 (0.7-0.7)
RR	42.7 (40.8-44.5)	80.4 (80.1-80.7)	8.0 (7.6-8.5)	97.2 (97.1-97.3)	2.2 (2.1-2.3)	0.7 (0.7-0.7)
SBP	37.3 (35.5-39.2)	80.4 (80.1-80.7)	7.7 (7.2-8.1)	96.7 (96.6-96.9)	1.9 (1.8-2.0)	0.8 (0.8-0.8)
**Empirically-derived vital signs with simplified age-based cutoffs**						
HR	46.4 (44.6-48.3)	76.5 (76.2-76.8)	7.4 (7.0-7.8)	97.3 (97.1-97.4)	2.0 (1.9-2.1)	0.7 (0.7-0.7)
RR	38.8 (37.0-40.6)	83.1 (82.9-83.4)	8.5 (8.0-9.0)	97.1 (97.0-97.3)	2.3 (2.2-2.4)	0.7 (0.7-0.8)
SBP	35.3 (33.5-37.1)	82.4 (82.1-82.7)	8.0 (7.5-8.5)	96.7 (96.5-96.8)	2.0 (1.9-2.1)	0.8 (0.7-0.8)

HR, heart rate; RR, respiratory rate; SBP, systolic blood pressure; Acc, accuracy; Sens, sensitivity; Spec, specificity; NPV, negative predictive value; PPV, positive predictive value; PLR, positive likelihood ratio; NLR, negative likelihood ratio

© 2024 Gorski JK et al. *JAMA Network Open.*

eTable 4. Areas Under the Receiver Operating Characteristic Curve (AUROCs) With 95% CIs for Multivariable Models in the Derivation (2021 TQIP) and Validation (2019 TQIP) Samples

	**AUROC in derivation sample (95% CI)**	**AUROC in validation sample (95% CI)**
PALS	69.6 (68.6-70.6)	69.4 (68.3-70.5)
ATLS	65.4 (64.4-66.3)	66.0 (64.9-67.0)
Empirically-derived	70.9 (69.9-71.8)	68.9 (69.9 -71.0)

PALS, Pediatric Advanced Life Support; ATLS, Advanced Trauma Life Support

© 2024 Gorski JK et al. *JAMA Network Open.*

eTable 5. Alternative Cutoffs for Vital Signs Obtained When Using Different Outcome Measures. Numbers in parenthesis represent 95% confidence intervals.

	Cribari Matrix			Need for Trauma Intervention			Need for Emergent Intervention within 6 hours		
	**HR**	**RR**	**SBP**	**HR**	**RR**	**SBP**	**HR**	**RR**	**SBP**
Z score for lower limit	-1.48	-1.00	-0.98	-1.05	-1.00	-1.05	-1.12	-1.01	-1.03
Z score for upper limit	0.82	1.27	1.55	1.07	1.31	1.28	1.11	1.30	0.96
0 months to <3 months	93 - 162	22 - 50	74 - 152	112 - 169	22 - 51	74 - 138	109 - 170	22 - 51	74 - 138
3 months to <6 months	103 - 159	23 - 46	81 - 134	117 - 166	23 - 47	80 - 126	115 - 167	23 - 47	80 - 126
6 months to <9 months	103 - 155	22 - 42	84 - 135	114 - 162	22 - 42	84 - 128	112 - 163	22 - 42	84 - 128
9 months to <1 year	100 - 155	22 - 40	86 - 137	111 - 162	22 - 40	85 - 130	109 - 164	22 - 40	85 - 130
1 year to <3 years	94 - 152	20 - 36	90 - 139	104 - 159	20 - 36	90 - 132	102 - 160	20 - 36	90 - 132
3 years to <6 years	83 - 132	18 - 30	94 - 136	92 - 139	18 - 30	94 - 130	91 - 140	18 - 30	94 - 130
6 years to <9 years	77 - 121	17 - 26	99 - 138	85 - 127	17 - 27	99 - 132	83 - 129	17 - 27	99 - 132
9 years to <12 years	74 - 117	16 - 24	103 - 142	81 - 122	16 - 25	103 - 137	80 - 124	16 - 25	103 - 137
12 years to <18 years	68 - 113	15 - 22	110 - 152	75 - 119	15 - 22	110 - 147	74 - 120	15 - 22	110 - 147

CM, Cribari Matrix; NFTI, Need for Trauma Intervention; NEI-6, Need for Emergent Intervention within 6 hours; HR, heart rate; RR, respiratory rate; SBP, systolic blood pressure.

© 2024 Gorski JK et al. *JAMA Network Open.*

eTable 6. Diagnostic accuracy of cutoffs (derived from different outcomes) in addition to PALS and ATLS criteria

**Cribari Matrix**						
	**Sens**	**Spec**	**PPV**	**NPV**	**PLR**	**NLR**
PALS						
HR	43.8 (42.6-45.0)	70.4 (70.0-70.7)	13.5 (13.0-13.9)	92.2 (92.0-92.5)	1.5 (1.4-1.5)	0.8 (0.8-0.8)
RR	40.2 (39.0-41.4)	75.8 (75.5-76.1)	14.9 (14.4-15.4)	92.3 (92.1-92.6)	1.7 (1.6-1.7)	0.8 (0.8-0.8)
SBP	60.1 (58.9-61.3)	42.9 (42.5-43.3)	10.4 (10.1-10.7)	90.7 (90.3-91.0)	1.1 (1.0-1.1)	0.9 (0.9-1.0)
ATLS						
HR	39.5 (38.3-40.7)	76.1 (75.8-76.5)	14.9 (14.3-15.4)	92.3 (92.0-92.5)	1.7 (1.6-1.7)	0.8 (0.8-0.8)
RR	9.3 (8.6-10.0)	96.3 (96.1-96.4)	20.8 (19.4-22.3)	91.0 (90.8-91.2)	2.5 (2.3-2.7)	0.9 (0.9-0.9)
SBP	5.4 (4.9-6.0)	99.4 (99.3-99.4)	48.1 (44.4-51.8)	90.5 (90.3-90.7)	8.4 (7.3-9.7)	1.0 (0.9-1.0)
Empirically-derived						
HR	37.4 (36.3-38.6)	77.7 (77.4-78.1)	15.1 (14.5-15.6)	92.2 (92.0-92.4)	1.7 (1.6-1.7)	0.8 (0.8-0.8)
RR	38.1 (36.9-39.3)	78.6 (78.3-78.9)	15.8 (15.2-16.3)	92.3 (92.1-92.6)	1.8 (1.7-1.8)	0.8 (0.8-0.8)
SBP	28.7 (27.6-29.8)	83.6 (83.3-83.9)	16.2 (15.5-16.8)	91.4 (91.2-91.6)	1.7 (1.7-1.8)	0.9 (0.8-0.9)
**Need for Trauma Intervention**						
	**Sens**	**Spec**	**NPV**	**PPV**	**PLR**	**NLR**
PALS						
HR	42.4 (41.4-43.3)	70.9 (70.6-71.3)	19.9 (19.4-20.4)	87.9 (87.6-88.1)	1.5 (1.4-1.5)	0.8 (0.8-0.8)
RR	35.1 (34.2-36.1)	75.9 (75.6-76.2)	19.9 (19.3-20.5)	87.3 (87.0-87.6)	1.5 (1.4-1.5)	0.9 (0.8-0.9)
SBP	63.6 (62.7-64.6)	43.7 (43.3-44.1)	16.9 (16.5-17.2)	87.0 (86.6-87.4)	1.1 (1.1-1.1)	0.8 (0.8-0.9)
ATLS						
HR	36 (35.1-36.9)	76.5 (76.1-76.8)	20.7 (20.1-21.3)	87.5 (87.2-87.8)	1.5 (1.5-1.6)	0.8 (0.8-0.8)
RR	8.0 (7.5-8.6)	96.4 (96.2-96.5)	27.4 (25.8-29.1)	86.0 (85.8-86.3)	2.2 (2.1-2.4)	1.0 (0.9-1.0)
SBP	4.7 (4.3-5.1)	99.5 (99.5-99.6)	63.2 (59.6-66.7)	85.3 (85.1-85.6)	9.6 (8.2-11.1)	1.0 (1.0-1.0)
Empirically-derived						
HR	34.9 (33.9-35.8)	75.2 (74.8-75.5)	19.3 (18.7-19.9)	87.2 (86.9-87.4)	1.4 (1.4-1.4)	0.9 (0.9-0.9)
RR	32.2 (31.3-33.1)	80.6 (80.3-81.0)	22.1 (21.4-22.7)	87.5 (87.2-87.8)	1.7 (1.6-1.7)	0.8 (0.8-0.9)
SBP	31.8 (30.9-32.7)	79.7 (79.4-80.0)	22.0 (21.3-22.7)	86.7 (86.4-87.0)	1.6 (1.5-1.6)	0.9 (0.8-0.9)
**Need for Emergent Intervention within 6 Hours**						
	**Sens**	**Spec**	**NPV**	**PPV**	**PLR**	**NLR**
PALS						

© 2024 Gorski JK et al. *JAMA Network Open.*

HR	37.7 (36.9-38.6)	70.4 (70.1-70.8)	21.4 (20.8-21.9)	84.2 (83.8-84.5)	1.3 (1.2-1.3)	0.9 (0.9-0.9)
RR	30.5 (29.6-31.3)	75.3 (74.9-75.7)	20.8 (20.2-21.4)	83.6 (83.2-83.9)	1.2 (1.2-1.3)	0.9 (0.9-0.9)
SBP	62.1 (61.2-63.0)	43.6 (43.2-44.0)	19.6 (19.2-20.1)	83.8 (83.4-84.3)	1.1 (1.1-1.1)	0.9 (0.8-0.9)
ATLS						
HR	30.3 (29.5-31.2)	75.7 (75.4-76.1)	21.0 (20.4-21.6)	83.6 (83.3-83.9)	1.2 (1.2-1.3)	0.9 (0.9-0.9)
RR	6.3 (5.9-6.8)	96.2 (96.0-96.3)	26.0 (24.5-27.6)	82.8 (82.5-83.1)	1.7 (1.5-1.8)	1.0 (1.0-1.0)
SBP	3.5 (3.2-3.9)	99.4 (99.3-99.5)	56.8 (53.1-60.5)	82.3 (82.0-82.6)	5.9 (5.1-6.9)	1.0 (1.0-1.0)
Empirically-derived						
HR	28.8 (28.0-29.6)	77.7 (77.4-78.0)	21.6 (21.0-22.2)	83.7 (83.4-84.0)	1.3 (1.3-1.3)	0.9 (0.9-0.9)
RR	28.1 (27.3-28.9)	79.4 (79.1-79.8)	22.5 (21.9-23.2)	83.8 (83.5-84.1)	1.4 (1.3-1.4)	0.9 (0.9-0.9)
SBP	39.1 (38.2-40.0)	69.4 (69.0-69.8)	22.1 (21.5-22.7)	83.7 (83.3-84.0)	1.3 (1.2-1.3)	0.9 (0.9-0.9)

PALS, Pediatric Advanced Life Support; ATLS, Advanced Trauma Life Support; Sens, sensitivity; Spec, specificity; NPV, negative predictive value; PPV, positive predictive value, PLR, positive likelihood ratio; NLR, negative likelihood ratio

© 2024 Gorski JK et al. *JAMA Network Open.*
