# Supplementary material for: Comparison of Vital Sign Cutoffs to Identify Children With Major Trauma
Source: JAMA Netw Open. 2024 Feb 16;7(2):e2356472. doi: 10.1001/jamanetworkopen.2023.56472 (PMC10873773; doi:10.1001/jamanetworkopen.2023.56472)
Supplement: Supplement 2. — Data Sharing Statement [file jamanetwopen-e2356472-s002.pdf]

Data Sharing Statement

Gorski. Comparison of Vital Sign Cutoffs to Identify Children With Major Trauma. *JAMA Netw Open*. Published February 16, 2024. doi:10.1001/jamanetworkopen.2023.56472

Data
Data available: No

Additional Information
Explanation for why data not available: The data used for this study are provided by the American College of Surgeons and may be obtained with a license at https://www.facs.org/quality-programs/trauma/quality/trauma-quality-improvement-program/
